# COPD prevalence and characteristics among sample of working population

**DOI:** 10.3389/fpubh.2025.1598290

**Published:** 2025-05-27

**Authors:** Sasho Stoleski, Jordan Minov, Dragan Mijakoski, Hana Brborović, Milan Milošević, Roko Žaja

**Affiliations:** ^1^Faculty of Medicine, Institute of Occupational Health of R. North Macedonia, WHO CC, GA2LEN CC, Ss. Cyril and Methodius, University in Skopje, Skopje, North Macedonia; ^2^Department of Environmental and Occupational Health and Sports Medicine, Andrija Štampar School of Public Health, University of Zagreb, School of Medicine, Zagreb, Croatia

**Keywords:** COPD, occupational exposure, noxious particles and gasses, pre-and post-bronchodilator spirometry, questionnaire

## Abstract

**Introduction:**

COPD is a global public health problem, causing a very high rates of morbidity, mortality, and work disability in the last decades worldwide.

**Objective:**

To determine the prevalence and characteristics COPD in a random sample of workers form the city of Skopje, and its relation to occupational exposures.

**Methods:**

A cross-sectional study was conducted including 1,867 workers (959 males and 908 females) from the city of Skopje. Afterwards, the study subjects were divided into exposed (1.287/68.9%) and unexposed (580/31.1%) groups based on their current job exposure to noxious particles and gasses. All study participants completed a questionnaire and underwent pre-and post-bronchodilator spirometry.

**Results:**

COPD prevalence was 3.9% among all workers. COPD prevalence in exposed workers was significantly higher compared to unexposed (4.7% vs. 2.4%). Significant difference was found in exposed workers with exposure duration longer than 20 years as compared to those with shorter duration of exposure (6.0 vs. 3.2%). COPD prevalence in workers who smoked was significantly higher than in non-smoking workers in both exposed (6.0% vs. 3.9%, *p* = 0.037) and unexposed (3.9% vs. 1.8%, *p* = 0.000) groups. The difference between workers with COPD in terms of use of solid and liquid bio fossil fuels at home and central heating/electricity is not significant (5.0% vs. 3.6%), both in exposed (5.4% vs. 4.3%), as well as non-exposed workers (3.8% vs. 1.9%).

**Conclusion:**

Our findings confirmed the role of occupational exposures in COPD prevalence indicating a need of more effective preventive activities in order to reduce the overall disease burden.

## 1 Introduction

The persistent and progressive airflow limitation within chronic obstructive pulmonary disease (COPD) is due to a combination of small airway disease and alveolar wall destruction, changes that occur to varying degrees in all COPD patients. Associated diseases (comorbidities), i.e., cardiovascular and metabolic diseases, osteoporosis and other disorders of the osteomuscular system, mental disorders, etc., have a significant impact on the morbidity and mortality of patients with COPD (1).

According to morbidity, disability, mortality and huge costs of national health systems, COPD has grown into one of the most important public health problems worldwide in the last few decades. Estimates based on the results of large epidemiological studies show that in 2010 about 300 million people worldwide have had COPD (2). The number of deaths from the disease in the same year is estimated at about 3 million people, and it is predicted that by 2060 the number of COPD deaths will rise to about 5.4 million. It is considered that the reasons for the great increase in the frequency of the disease in developing countries is the increase in the frequency of smoking, and in developed countries the aging of the population (2, 3).

Having in mind the results of epidemiological studies performed in the last two decades in Europe, United States, and Australia, the frequency of COPD in the general adult population is 4–12%. Although the frequency of the disease in women is increasing, it is more common in men. The frequency of COPD increases with age, so that in the age group over 45 years. The disease is statistically significantly more common compared to its frequency in the age group younger than 45 years (3–5).

It is believed that COPD occurs as a result of a complex interaction between endogenous factors and factors from the external environment. The most important endogenous factors in the occurrence of the disease are: genetic factors, gender, age, lung development in the fetal period and childhood, respiratory infections in childhood, chronic bronchitis, etc. The most important exogenous factors in the occurrence and progression of COPD are: exposure to harmful particles and gasses, diet, socioeconomic status, etc. (1). Exposure to harmful particles and gasses is of primary importance in the onset and progression of the disease. Smoking, both active and passive, is the most important and best-studied risk factor for the development of COPD. Tobacco smoke is a complex mixture of particles and gasses containing about 4,000 identified and an unknown number of unidentified substances. For about 250 of them, it has been proven that they have an irritating and toxic effect, and for about 60 that they have a proven or probable carcinogenic effect on various organs and systems of the human body. COPD occurs in about 15–20% of active smokers, and, at the same time, 60–70% of patients with COPD are current or former smokers (5, 6).

According to current knowledge, one of the most important pathogenetic mechanisms of COPD is the disturbed balance between proteinases and antiproteinases in the lungs with the dominance of proteinase activity. Increased activity of proteinases released by macrophages and neutrophils is responsible for destruction of alveolar walls and loss of elastin from lung tissue. At the same time, proteinases are potent stimulators of mucus secretion in the airways. On the other hand, a significant percentage of all COPD cases occur in people who have never smoked or been exposed to tobacco smoke from other smokers. Occupational and environmental aeropollutants, especially indoor aeropollutants released during the combustion of biofuels, are a significant risk factor for the development of the disease. The risk is particularly high among people who work in certain occupations and are active or passive smokers at the same time (6–10).

The results of several studies carried out in recent decades suggest that occupational agents play a role in the development of COPD and its progression independent of the effect of tobacco smoke and aging, as well as in the occurrence of exacerbations of the disease (10, 11).

In the last three decades, several studies have been performed investigating the role of occupational agents in the development of COPD. According to the 2019 American Thoracic Society (ATS) Report about the role of occupational exposure in the occurrence of lung diseases, occupational agents participate in the occurrence of 10–20% of all cases with COPD, that is, in one out of five patients with COPD, occupational exposure is the cause for the disease. According to the same source, 60–70% of all COPD cases are due to the effects of tobacco smoke, in about 20% of smoking patients, that is, in 50–60% of non-smoking patients, the disease is due to the effects of occupational agents, and in about 10% the effects of indoor environmental pollutants, primarily the smoke released from biofuels used for household cooking and heating. On the other hand, it is considered that the role of occupational agents in the occurrence of COPD in developing countries is much greater in relation to their role in developed countries, i.e., in countries with much higher standards of safety and health protection at work (1, 2). Occupational agents that have been shown to cause COPD in predisposed workers are: dust containing free silica, coal dust, cotton dust, wood dust, grain dust, dust to which crop and dairy farmers are exposed, cadmium dust and smoke, welding fumes, sulfur dioxide, nitrogen oxides, diesel particles, etc. On the other hand, job positions with an increased risk of developing COPD are: construction workers (bricklayers, facade workers, terracers, etc.), roads and tunnels construction workers, miners (coal mines, metal mines), workers in metallurgy, textile workers, farmers, welders, wood industry workers, traffic workers, etc. The risk of developing COPD in predisposed non-smoking workers from dusty occupations is high, but, as previously stated, it is even higher among smokers from those occupations. The risk of the interaction of tobacco smoke and occupational exposure at individual workplaces is not additive, but multiplicative (11–14).

The aim of the present study was to determine the COPD prevalence in a sample of working population and to classify the disease according to the degree of its severity. Also, the aim was to determine the disease distribution among working population subjects according to their gender, age, exposure to occupational factors of interest and length of exposure, smoking status, family history of asthma/chronic bronchitis, and mode of household heating and cooking, as well as to determine the workplaces with the highest frequency of COPD.

## 2 Methods

### 2.1 Study design and setting

A cross-sectional epidemiological study (prevalence study) was carried out at the Institute for Occupational Health of Republic of North Macedonia, Skopje, in the period 2018–2021. The study was performed within the scientific projects of all departments of the Faculty of Medicine, Ss. Cyril and Methodius University in Skopje, based on the Decision of the Faculty Management, and approved by the Ethics Committee of the Institute for Occupational Health of R. North Macedonia, Skopje for conducting the study and publishing of the obtained results (0302-236/2018). All study subjects were informed about the study and gave their written consent.

### 2.2 Study population

The study population included 1,867 active workers (959 males and 908 females, aged 18–67 years) from the Skopje region recruited during their preventive medical examinations at the Institute for Occupational Health of R. North Macedonia - Skopje. In order to be representative, study sample was calculated by the software program PEPI 4.04, with 95% confidence level and confidence interval ± 5. The actual study was a part of a larger survey on COPD prevalence and characteristics in a sample of general adult population from the Skopje Region which included 2.348 participants (active workers, retired persons, and students) (14).

### 2.3 Study protocol

The study was performed following the actual recommendations of European Respiratory Society (ERS) and American Thoracic Society (ATS) for epidemiological studies on COPD (15).

The study protocol included completion of a questionnaire and spirometric measurements. An interviewer-led questionnaire was based on two standardized questionnaires, i.e., Population-based screening questionnaire for COPD and Symptom-based questionnaire for identifying COPD, and it consisted of three parts (16, 17).

The first part included questions on demographics of the study subjects, personal and family history of chronic bronchitis and asthma, the fuels used for heating, cooking and other household needs, as well as questions on actual or/and previous occupational exposures. Occupational exposures in the working population were assessed also by the Risk assessment report of the company in which they were employed. Also, we have used certain questions regarding workplace exposure regarding occupational exposure to: (1) dust, gasses, fumes, and vapors; (2) exposure to physical exertion; (3) exposure to high/low temperatures; (4) duration of exposure; and (5) smoking habits (smoker, non-smoker, and ex-smoker).

The second part included questions on smoking status of the study subjects. The smoking status (active smoker, ex-smoker, and non-smoker) was defined by the World Health Organization (WHO) criteria (18).

The third part of the questionnaire included questions on respiratory symptoms in the last 12 months (nasal symptoms, cough, phlegm, dyspnea, wheezing, and chest tightness). In the subjects with dyspnea, its severity was assessed according to the criteria of Modified British Medical Council (mMRC) (19).

The study protocol did not include data about residential air pollution exposure or data about workplace air quality measurements, and therefore there is no analysis of the relationship between COPD prevalence and pollution levels.

### 2.4 Baseline and post-bronchodilator spirometry

Spirometric measurements included baseline (pre-bronchodilator) spirometry which was performed in all study subjects, and post-bronchodilator spirometry which was performed in subjects with value of the ratio between forced expiratory volume in 1 s (FEV1) and forced vital capacity (FVC) less than 0.70. The baseline spirometry, including measures of FVC, FEV1, FEV1/FVC, and maximal expiratory flow at 75, 50, 25%, and 25–75% of FVC (MEF75, MEF50, MEF25, and MEF25-75, respectively), was performed in all subjects using spirometer Ganshorn SanoScope LF8 (Ganshorn Medizin Electronic GmbH, Germany) with recording the best result from three measurements the values of FEV1 of which were within 5% of each other.

Spirometry measurements were performed by experienced and properly trained health professionals supervised by physicians who spent at least 2 years of training in the field, whereas spirometer was calibrated on a weekly basis following recommended calibration procedures and quality control measures given by the manufacturer.

The results of spirometry were expressed as percentages of the predicted values according to the actual recommendations, protocols and guidelines given by the European Respiratory Society and the American Thoracic Society. The post-bronchodilator spirometry was performed according to the actual recommendations, i.e., spirometric measurements were performed 20 min after administration of 400 μg salbutamol by metered dose inhaler through spacer. Fixed airflow narrowing characteristic for COPD was considered if post-bronchodilator FEV1/FVC remained less than 0.70 (20–22).

### 2.5 Definition and classification of COPD

The existence of COPD is determined according to the presence of symptoms in subjects in whom a persistent decrease in air flow through the airways has been proven by spirometry, i.e., a post-bronchodilator value of the FEV1/FVC ratio lower than 0.7. According to the degree of severity of the spirometric impairment, subjects with COPD are classified into four groups: mild COPD or GOLD 1 (FEV1 value equal to or higher than 80% of the predicted value), moderate COPD or GOLD 2 (FEV1 value from 50 to 80% of predicted value), severe COPD or GOLD 3 (FEV1 value from 30 to 50% of the predicted value) and very severe COPD or GOLD 4 (FEV1 value lower than 30% of the predicted value) (1, 15).

### 2.6 Statistical analysis

Statistical analysis was performed using the Statistical Package for the Social Sciences (SPSS), version 11.0 for Windows. Continuous variables were expressed as mean values with standard deviation (SD), and the nominal variables as numbers and percentages. In line with the aim of the study, for analyses of the data we used univariate statistical models for testing the differences in prevalence and comparison of the means. Chi-square test (or Fisher’s exact test where appropriate) was used for testing difference in the prevalence. Comparison of spirometric measurements was performed by independent-samples *T*-test. A *p*-value less than 0.05 was considered as statistically significant.

## 3 Results

### 3.1 Demographics of the study subjects

The study includes a total of 1,867 subjects from the working population, of which 959 (51.4%) are men and 908 (48.6%) are women, aged 18–67 years. Regarding occupational exposure, 1,287 (68.9%) subjects from the working population (713 men and 574 women) are exposed to dust, gasses, fumes and vapors, physical exertion and/or low temperatures, while 580 (31.1%) subjects (246 men and 334 women) are not exposed to these occupational hazards (Table 1).

**Table 1:** Distribution of working population subjects according to workplace and occupational exposure of interest.

Workplace and occupational exposure	Number of subjects (*N* = 1,867)
**Non-exposed workers**	**580 (31.1%)**
Administrative workers (lawyers, economists, archivists, accountants, etc.), teachers, IT sector, etc.	
**Exposed workers**	**1,287 (68.9%)**
Construction workers (inorganic dust containing free silica, physical exertion, low temperatures)	114 (6.1%)
Professional drivers (diesel particles, low temperatures)	94 (5.1%)
Farmers/cereal crops (grain dust, physical exertion, low temperatures)	87 (4.6%)
Workers in the production of paints and varnishes (organic solvents)	87 (4.6%)
Cattle breeders/cow farmers (organic dust, physical exertion, low temperatures)	83 (4.4%)
Healthcare workers (disinfectants, latex)	78 (4.1%)
Textile workers (cotton dust, physical exertion)	73 (3.9%)
Electricians (inorganic dust, physical exertion, low temperatures)	68 (3.6%)
Bakers/millers (flour dust, physical exertion)	63 (3.3%)
Production of herbal teas (organic dust, physical exertion)	59 (3.2%)
Hygienists (cleaning and disinfecting agents, physical exertion, low temperatures)	58 (3.1%)
Workers in the chemical industry (organic solvents)	47 (2.5%)
Metal workers and welders (metal dust, gasses and fumes, welding fumes)	43 (2.3%)
Furniture manufacturing workers (wood dust, physical exertion, low temperatures)	37 (1.9%)
Other workers (hairdressers, beauticians, car mechanics, car painters, firefighters, painters, tinsmiths, cooks, salesmen, electricians, plumbers, security guards, printing workers, security workers, etc.)	296 (15.8%)

Data are given as number and percent of subjects with certain variable.

All workers are exposed to occupational hazards of interest such as various types of dust, gasses, fumes or vapors, i.e., they work on the so-called “dusty occupations” or “dusty trades,” so further on the term “exposed workers” will refer to them. Part of the working population, in addition to exposure to dust, gasses, fumes or vapors, are exposed to one or both of the occupational hazards of interest. Three hundred and eighty-four subjects (20.6% of the working population, i.e., 29.8% of the exposed workers) were exposed to physical exertion, and 288 subjects (17.1% of the working population, i.e., 22.4% of the exposed workers) were exposed to low temperatures.

Regarding the length of work experience, that is, the duration of exposure to the mentioned occupational hazards, 598 subjects (46.4%) were exposed for less than 20 years, and 689 (53.6%) for more than 20 years. Having in mind the length of work experience among non-exposed workers, 266 subjects (45.8%) have work experience less than 20 years, and 314 subjects (54.1%) have work experience greater than 20 years.

The frequency of smokers among the working population is 38.3%, that is, 41.4% among workers exposed to occupational hazards and 31.6% among workers who are not exposed to occupational hazards of interest. A positive family history of asthma/chronic bronchitis was registered in 22.1% of the subjects, 22.5% in exposed and 19.8% in non-exposed workers. Regarding the method of heating and cooking at home, solid and liquid biofuels are used by 28.9% of the subjects, 29.8% of the exposed and 26.7% of the non-exposed workers.

### 3.2 Prevalence and characteristics of COPD among the examined subjects

COPD is registered among 3.9% of the working population subjects. The difference in the disease frequency in males (4.4%) and females (3.5%) of the working population subjects is not statistically significant.

**Figure 1:**
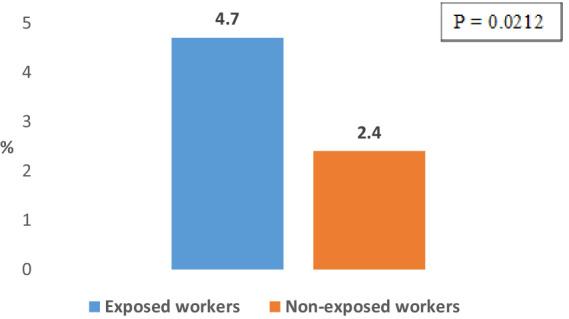
Prevalence of COPD in working population subjects exposed and non-exposed to dusts, gasses, vapors, and fumes.

The frequency of COPD among the working population subjects exposed to dusts, gasses, fumes and vapors, with or without exposure to physical exertion and/or low temperatures, (4.7%) is significantly higher compared to its frequency among working population subjects who are not exposed to these occupational hazards (2.4%) (*p* = 0.0212) (Figure 1). At the same time, 53.3% of the exposed workers with COPD were smokers. The difference in the prevalence of COPD between males and females was not statistically significant in either exposed (5.1% vs. 4.2%) or unexposed workers (2.8% vs. 2.2%).

The difference in the COPD frequency between subjects from the working population occupationally exposed to physical exertion, with or without exposure to other occupational hazards of interest, (5.9%) and subjects from the working population who are not exposed to physical exertion (5.2%) is not statistically significant.

Also, the difference in the frequency of COPD between subjects from the working population occupationally exposed to low temperatures, with or without exposure to other occupational hazards of interest (5.5%) and subjects from the working population who are not exposed to this occupational hazard (4.4%), is not statistically significant.

**Figure 2:**
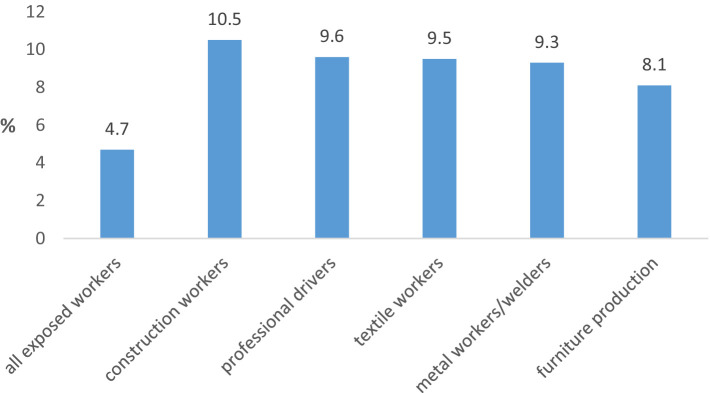
Exposed workers with the highest COPD prevalence.

The highest COPD prevalence among exposed workers was registered in construction workers (10.5%), professional drivers (9.6%), textile workers (9.5%), metal workers and welders (9.3%) and furniture production workers (8.1%) (Figure 2). The frequency of the disease in these groups of workers was significantly higher than in other groups of exposed workers being within the range 3 to 6%.

**Figure 3:**
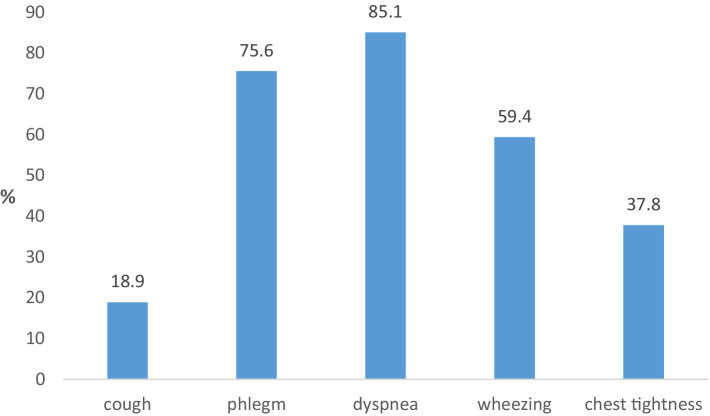
Frequency of respiratory symptoms in the last 12 months in working population subjects with COPD.

The most common respiratory symptoms among working population subjects with COPD are dyspnea (85.1%) and cough with phlegm (75.6%) (Figure 3). No significant difference was registered in the frequency of individual symptoms in exposed and non-exposed subjects from the working population.

The mean post-bronchodilator values of the basic spirometric parameters of subjects with COPD from the working population are shown in Table 2.

**Table 2:** Mean post-bronchodilator values of the basic spirometric parameters in the working population subjects with COPD.

Spirometric parameters	Mean post-bronchodilator values (% of predicted value)
FVC (% pred)	80.8 ± 14.1
FEV1 (% pred)	59.2 ± 9.1
FEV1/FVC	0.66 ± 0.02

Numerical data are expressed as mean value with standard deviation.

FVC: forced vital capacity; FEV_1_: forced expiratory volume in 1 s; % pred: % of predicted value.

Mean post-bronchodilator values of basic spirometric parameters in exposed workers with COPD are significantly lower compared to mean post-bronchodilator values of basic spirometric parameters in non-exposed workers with COPD (Table 3).

**Table 3:** Mean post-bronchodilator values of basic spirometric parameters in exposed and non-exposed workers with COPD.

Spirometric parameters	Exposed workers with COPD (*N* = 60)	Non-exposed workers with COPD (*N* = 14)	*P*-value*
FVC (% pred)	77.3 ± 9.6	83.9 ± 11.6	0.016
FEV1 (% pred)	55.4 ± 7.1	61.7 ± 9.7	0.003
FEV1/FVC	0.64 ± 0.03	0.67 ± 0.01	0.000

Numerical data are expressed as mean value with standard deviation.

FVC: forced vital capacity; FEV_1_: forced expiratory volume in 1 s; % pred: % of predicted value.

Tested by *t*-test for independent variables.*Statistical significance.

According to the degree of spirometric impairment, the majority of the working population subjects with COPD are classified in the GOLD 1 and GOLD 2 groups (mild and moderate COPD) (Table 4). The difference in the distribution of exposed and non-exposed working population subjects with COPD is not statistically significant.

**Table 4:** Distribution of working population subjects with COPD according the degree of spirometric impairment.

GOLD stadium (degree of spirometric impairment)	Working population subjects with COPD (*N* = 74)	Exposed workers (*N* = 60)	Non-exposed workers (*N* = 14)	*P*-value*
GOLD 1 (FEV1 > 80%)	35 (47.2%)	27 (45%)	8 (57.1%)	*P* > 0.05
GOLD 2 (FEV1 = 50 to 80%)	29 (39.2%)	24 (40%)	5 (35.7%)	*P* > 0.05
GOLD 3 (FEV1 = 30 to 50%)	9 (12.2%)	8 (13.2%)	1 (7.1%)	*P* > 0.05
GOLD 4 (FEV1 < 30%)	1 (1.6%)	1 (1.6%)	/	*P* > 0.05

Data are given as number and percent of subjects with certain variable.

* Tested by chi-square test or Fisher’s exact test where appropriate.

**Figure 4:**
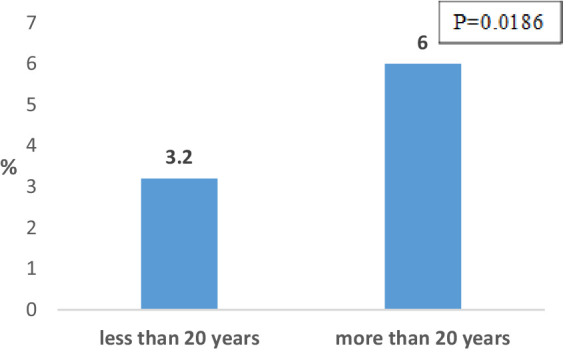
Distribution of exposed workers with COPD according to exposure duration.

The frequency of COPD among working population subjects with work experience greater than 20 years (4.9%) is significantly higher compared to those with shorter work experience (2.8%) (*p* = 0.0447). The frequency of COPD among exposed workers with exposure duration of more than 20 years. is significantly higher in relation to its frequency among exposed workers with shorter exposure duration (6.0% vs. 3.2% *p* = 0.0186) (Figure 4). The difference in the frequency of COPD among non-exposed workers with work experience greater than 20 years (2.5%) and younger than 20 years (2.2%) is not significant.

**Figure 5:**
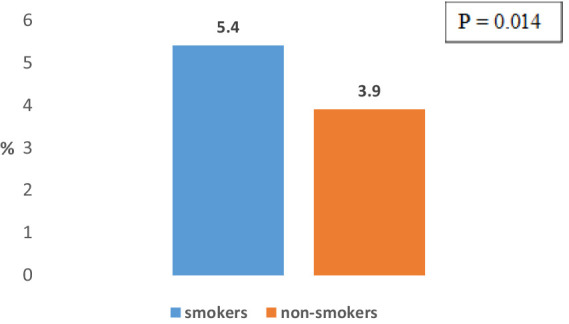
Distribution of working population subjects with COPD according smoking status.

According to the smoking status, the frequency of COPD among working population subjects who smoke (5.4%) is significantly higher compared to its frequency among those who do not smoke (3.9%) (*p* = 0.014) (Figure 5).

**Figure 6:**
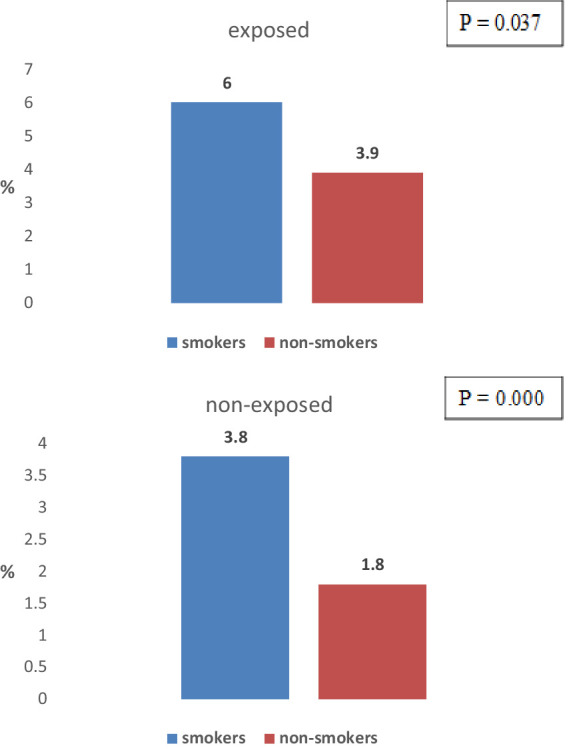
Distribution of COPD in exposed and non-exposed workers according smoking status.

The difference in the frequency of COPD between exposed workers who smoke (6%) and exposed workers who do not smoke (3.9%) is statistically significant (*p* = 0.037) (Figure 6).

**Figure 7:**
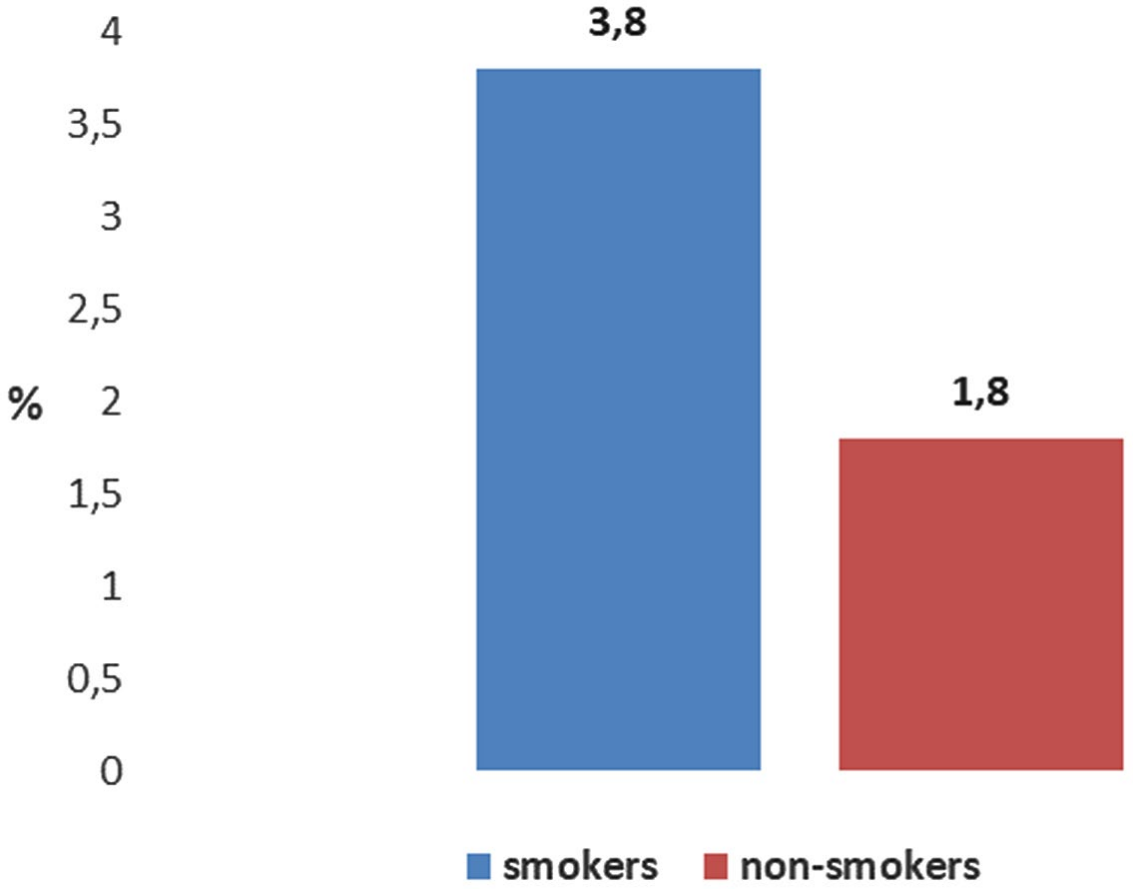
Distribution of COPD among non-exposed workers according smoking status.

The difference in the frequency of COPD between non-exposed workers who smoke (3.8%) and non-exposed workers who do not smoke (1.8%) is statistically significant (*p* = 0.000) (Figure 7).

No significant difference was registered in the frequency of COPD between working population subjects who have a positive family history of asthma/chronic bronchitis (4.3%) and those with a negative family history of these diseases (3.8%). The difference in the frequency of COPD in exposed and unexposed workers with positive and negative family history of asthma/chronic bronchitis is also not significant (4.8 and 4.6% in exposed and 2.4 and 2.4% among non-exposed workers).

The difference within the working population subjects with COPD regarding the use of solid and liquid bio-fossil fuels in homes and central heating/electricity is not significant (5.0 and 3.6%), both in exposed workers (5.4 and 4.3%), as well as among the non-exposed workers (3.8 and 1.9%).

## 4 Discussion

The results of the current study confirm the association of COPD with the occupational exposure among subjects. Namely, the frequency of COPD in subjects who are exposed to dust, gasses, vapors and fumes is about two times higher in relation to its frequency in unexposed subjects, while in both cases no statistically significant difference in the frequency of the disease was registered between male and female subjects. Also, a statistically significantly higher frequency of COPD was registered among the exposed subjects with more than 20 years of work experience compared to its frequency among exposed subjects with shorter working experience. The highest frequency of COPD among subjects from certain job positions (about 10%) was registered among construction workers, professional drivers, textile workers, metal workers (welders) and workers in furniture production. Similar results were obtained in our previous workplace-based studies (23–26), as well as in population and workplace-based surveys conducted in other countries (7, 11).

COPD is one of the most common chronic non-communicable diseases in the world in recent decades with huge financial implications for national health systems, one of the most common causes of death, and also one of the most common causes of reduced work ability and early retirement in the modern world. The results of research in this area show that the problem of COPD continues to grow and generate huge costs, which is especially pronounced in developing countries. It is assumed that there are about 100,000 people with COPD in the Republic of North Macedonia, but until now, as in many other countries, no epidemiological research has been performed at the level of a working population (population-based study) (5, 14). In the last decade, several studies have been carried out in our country in which workers from certain workplaces were included (workplace-based study) and in which the frequency of the disease in workers with occupational exposure, which is considered a risk factor for the occurrence and progression of COPD, was compared with its frequency among workers who are not exposed to those occupational hazards (23–32). In the current research of the type of population study, the first of its kind in our country, 1,867 workers were included. The research methodology follows the recommendations for performing this type of study on COPD, that is, it consists of filling out a questionnaire (interviewer-led questionnaire) and spirometry for all subjects with a bronchodilator test for subjects with reduced lung function (spirometrically defined COPD). The study includes a working population, and depending on the exposure to the occupational hazards of interest (dust, gasses, vapors and fumes, physical exertion and low temperatures), it consists of exposed and non-exposed subjects.

Among working population, a high frequency of active smokers was registered (about 38%), which is in the same range as their frequency in neighboring and Mediterranean countries, and much higher than the frequency of active smokers in the countries of Western and Northern Europe and North America (9). At the same time, a low frequency of ex-smokers was registered (about 6%), which corresponds to the results obtained from our previous research (9, 33). The high frequency of active smokers and the low frequency of ex-smokers indicate insufficiently effective anti-smoking activities and the need for their improvement.

Regarding the frequency of respiratory symptoms in the last 12 months, the highest frequency is registered for cough, and the frequency of respiratory symptoms in the last 12 months is higher among exposed respondents from the working population compared to non-exposed ones. The obtained results are similar to the results registered in our previous research, as well as in the research in this area performed in other countries (30).

The frequency of COPD in the working population is 3.9% and is slightly higher in men compared to women, but the difference is not statistically significant. A statistically significant difference (4 times higher) was registered between workers older than 45 years and those younger than 45 years and, that is, among respondents from the age group older than 45 years.

Results on the prevalence of COPD obtained from population studies vary widely. Thus, in the research on the frequency of the disease in the adult population older than 40 years from several cities in Latin America, reported COPD prevalence ranged from 7.8% in Mexico City to 19.7% in Montevideo (34). According to the results of the BOLD (Burden of Obstructive Lung Diseases) survey of adults older than 40 years in 29 mostly European countries in which a standard methodology was applied, i.e., a questionnaire for respiratory symptoms with pre-and post-bronchodilator spirometry, the frequency of the disease in stages GOLD 2–4 is 10.1%, and its frequency in non-smokers ranges from 3 to 11% (35). The wide variation in the prevalence of COPD is due to a number of reasons. The results obtained from the population studies depend on the structure of the included subjects, that is, on their number, age, working status, smoking status, etc., as well as on the applied research methodology (questionnaire-based studies, questionnaire-based studies and spirometric tests, COPD diagnosed by a doctor, etc.). A serious lack of research is the absence of criteria for epidemiological diagnosis of COPD, which was corrected in the last decade with the recommendations for performing these studies, that is, with the recommendations for the application of a standardized questionnaire in combination with pre-and post-bronchodilator spirometry. On the other hand, the epidemiology of COPD is complex and variable with real geographical, temporal and other variations at the level of individual countries or individual regions within a country, e.g., differences in the frequency of smoking, dominant industrial branches, outdoor and indoor air pollution, etc. (36, 37).

Regarding the distribution of the disease according to the degree of its severity, the largest number of workers with COPD fall into the categories of mild and moderately severe disease. The distribution of the disease according to the degree of its severity differs between exposed and unexposed subjects of the working population. Namely, the average values of the basic spirometric parameters in exposed workers with COPD are statistically significantly lower than their average values in unexposed workers with COPD, and in the group of exposed workers with COPD, subjects with moderate and severe disease are more common compared to unexposed workers with COPD, but the difference is not statistically significant. Similar results have been obtained in our previous research involving workers occupationally exposed to dust, gasses, vapors and fumes and office workers, as well as in research conducted in other countries (14, 23–30).

Tobacco smoke, i.e., active smoking, is the most important and until now the best studied risk factor for the occurrence and progression of COPD with a proven dose–response relationship. The results of numerous studies show that cigarette smoking is associated with a higher frequency of respiratory symptoms, more frequent lung function impairment, a higher annual decrease in FEV1, and a higher mortality rate compared to non-smokers (33). It is considered that exposure to tobacco smoke is responsible for the occurrence of about two-thirds of all cases of COPD (tobacco-induced COPD), both in active and passive smoking (38). The results of the current research indicate that the COPD prevalence in smoking subjects is more than four times higher in relation to its frequency in non-smoking subjects. The frequency, on the other hand, of COPD among subjects with smoking experience greater than 20 years is higher compared to those with a shorter smoking experience, but the difference just missed statistical significance.

Although tobacco smoke is considered the most important risk factor for the development of COPD, the development of the disease related to exposure to tobacco smoke is associated with about two-thirds of all cases of COPD, and sometimes less. A significant percentage of all COPD cases also occur in people who have never smoked, indicating both the effects and other factors in the disease development. The results of several studies carried out in the last two decades indicate the importance of occupational exposure to dusts, gasses, vapors and fumes in the occurrence and progression of COPD, and for some occupational agents the mechanisms of the occurrence of anatomical and functional disorders characteristic of COPD have been clarified (e.g., silica dust-induced COPD) (39). The results of different studies on the frequency of COPD in workers from various occupations have large variations that are due to the previously mentioned factors, as well as to differences in the nature and degree of exposure to individual occupational agents in different countries and regions, existing occupational safety and health legislation in different countries, etc. (40).

The results of the current research indicate that the frequency of COPD in exposed workers is statistically significantly higher than in exposed workers who do not smoke. The results of longitudinal research indicate that the risk of COPD among workers in dusty occupations is about twice as high as the risk of the disease among workers from other (“non-dusty”) occupations, the risk of COPD among workers-smokers who are not occupationally exposed to dusts, gasses, vapors and fumes is about seven times higher than the risk of non-exposed non-smoking workers, while the risk of COPD in smoking workers from dusty occupations is about 14 times higher than the risk in unexposed non-smoking workers (41). Also, the results of several studies indicate that, considering the combined effect of smoking and occupational agents, preventive activities regarding COPD among workers in dusty occupations should be directed at both smoking and occupational exposure, since the fact that activities directed at one of these two factors do not have a significant impact in disease prevention (42, 43). Gender, genetic factors and exposure to harmful agents and gasses from the environment, primarily indoor air pollutants released during the combustion of solid and liquid biofuels, are considered risk factors for the development of COPD (1, 3, 4). According to the results of our research, the frequency of the disease is higher in men than in women, but the difference is not statistically significant. Also, no statistically significant difference was registered between subjects with a positive family history of asthma/chronic bronchitis and those with a negative family history of these diseases, nor between subjects who use solid and liquid biofuels for heating and cooking at home and those who use other sources for these purposes.

The results obtained in our current research should be interpreted keeping in mind its limitations and strengths. The distribution of subjects in individual job positions, is not even, which may affect the obtained results. Also, the registered frequency of COPD in exposed subjects may be lower due to the so-called “healthy workers’ effect,” that is, workers with respiratory symptoms often leave these hazardous workplaces. The data on the occupational exposure among the working population are based mainly on the questionnaire and data from the Safety Statement, without qualitative and quantitative assessment, and workplace air quality measurements. Finally, the research was also affected by the restrictive measures adopted due to the Covid-19 pandemic concerning stop of spirometry testing after the Covid-19 outbreak. Advantages of the research, however, are that it is the first population study on COPD in our country, and the sample is large enough to obtain relevant results, as well as the fact that the study was performed according to the current recommendations for studies on COPD prevalence (application of a questionnaire with pre-and postbronchodilator spirometry). Our findings confirmed the role of occupational exposures in COPD prevalence indicating a need of more effective preventive activities in order to reduce the overall disease burden.

## Data Availability

The data analyzed in this study is subject to the following licenses/restrictions: The raw data supporting the conclusions of this article will be made available by the authors, without undue reservation. Requests to access these datasets should be directed to dmijakoski@yahoo.com.
